# Systematic and Multi-Omics Prognostic Analysis of Lysine Acetylation Regulators in Glioma

**DOI:** 10.3389/fmolb.2021.587516

**Published:** 2021-02-26

**Authors:** Zewei Tu, Lei Wu, Haitao Luo, Jingying Li, Shigang Lv, Minhua Ye, Feng Liu, Chuming Tao, Xingen Zhu, Kai Huang

**Affiliations:** ^1^ Department of Neurosurgery, The Second Affiliated Hospital of Nanchang University, Nanchang, China; ^2^ East China Institute of Digital Medical Engineering, Shangrao, China; ^3^ Institute of Neuroscience, Nanchang University, Nanchang, China; ^4^ Department of Comprehensive Intensive Care Unit, The Second Affiliated Hospital of Nanchang University, Nanchang, China; ^5^ Department of Neurosurgery, Jiangxi Provincial Children’s Hospital, The Affiliated Children's Hospital of Nanchang University, Nanchang, China

**Keywords:** glioma, lysine acetylation regulator, epigenetic, prognostic signature, biomarker

## Abstract

Lysine acetylation modification, which has key roles in cellular homeostasis as well as cancer malignancy, is dynamically regulated by lysine acetylation regulators (LARs). In our study, we found that most of 33 evaluated LARs were differentially expressed among 1,125 gliomas grouped by different clinicopathological characteristics. Consensus clustering was applied to 33 LARs, resulting in three glioma subtypes (LA1, 2, and 3). The LA3 subgroup was associated with the poorest clinical outcome, higher WHO grade, fewer isocitrate dehydrogenase mutations, and lower frequency of 1p/19q codeletion. Furthermore, gene set enrichment analysis indicated that eight tumor hallmarks were highly enriched in the LA3 subgroup. These results suggested that LARs are significantly related to glioma malignancy. We then designed a LAR-signature based on 14 overall survival (overall survival)-related LARs, and showed that the LAR-signature possesses strong and independent prognostic value for glioma patients in both training and validation datasets. Moreover, by interrogating single nucleotide polymorphism and copy number variation (CNV) data in The *Cancer* Genome Atlas dataset, we found that higher score of our risk signature is correlated with the hypermutation status of gliomas and that HDAC1(1p) was one of the oncogenes lost in 1p/19q codeletion events, while SIRT2(19q) and EP300(22q) may act as tumor suppressors in gliomas with 19q or 22q deletions, respectively. In conclusion, LARs are critical for the malignant development of gliomas, and our results are useful for prognostic stratification and development of novel assessment strategies for the prognosis of glioma patients.

## Introduction

Glioma, the most common and fatal intracranial primary tumor in adults, is known for its rapid progression, high infiltration rate, and relative resistance to chemoradiotherapy (Cancer Genome Atlas Research Network et al., 2015; Jiang et al., 2016). Although comprehensive integrated treatment programs are currently available, the clinical outcomes for glioma patients remain poor (Cancer Genome Atlas Research Network, 2008; Cancer Genome Atlas Research Network et al., 2015; Jiang et al., 2016). According to the Chinese Glioma Genome Atlas (CGGA), patients with malignant glioma have a dissatisfactory prognosis with median overall survival (OS) of 78.1 months for low-grade gliomas (LGGs; WHO grade II), 37.6 months for anaplastic gliomas, and 14.4 months for glioblastomas (GBMs) (Jiang et al., 2016). In recent years, numerous glioma neuropathological biomarkers and molecular stratification of glioma patients have been identified based on the rapid development of biomedical and bioinformatics technology. However, the identification of new and efficient prognostic and therapeutic biomarkers and targets in clinical use remains a priority for glioma-tailored prognostic assessment and treatment.

Epigenetic regulation is essential for cellular homeostasis and its dysregulation is associated with a variety of cancers (Dawson and Kouzarides 2012; Henikoff and Greally 2016; Chen et al., 2017). Post-translational modifications (PTMs) are key elements of epigenetic regulation and function as signaling markers within oncocytes (Serrano-Gomez et al., 2016; Toh et al., 2017). Lysine acetylation is a dynamic, reversible PTM that has been widely investigated in recent years due to its ubiquity as a mechanism for cellular protein modification that regulates numerous cellular biological processes, including transcription, cell cycle, cell division, DNA damage repair, cellular signaling transduction, protein folding and aggregation, cytoskeleton organization, RNA processing and stability (Narita et al., 2019; Gil et al., 2017). Both histone and non-histone proteins, such as p53, STAT proteins, NF-κB, FoxO proteins, and tubulins, are targeted by lysine acetylation regulators (LARs), and several are the products of oncogenes or tumor-suppressor genes and are directly involved in tumorigenesis, tumor progression, and metastasis (Glozak et al., 2005; Narita et al., 2019).

Lysine acetylation is dynamically regulated by acetyltransferases and deacetylases. The main LARs comprise of acetyltransferase families, including the GCN5 family (KAT2A and KAT2B), p300 family (KAT3A [CREBBP] and KAT3B [EP300]), MYST family (KAT5, KAT6A, KAT6B, KAT7, and KAT8), and others, such as the SLC16A10, KAT1 (HAT1), ESCO1, and ESCO2; and deacetylase families, including the histone deacetylase family (HDAC1–11), Sirtuin deacetylase family (SIRT1–7), and others, such as the TCF1 (HNF1A) and LEF1 (Narita et al., 2019). Increasing evidence supports that LARs directly or indirectly participate in cancer initiation and progression, which led us to explore the roles of acetylation in glioma in greater detail. Although numerous studies have investigated the acetylation-related molecular regulatory mechanisms in gliomas, the role of lysine acetylation in glioma is still poorly understood, and clarifying the effects of impaired regulation of lysine acetylation could pave the way for new therapeutic approaches to treat patients with these diseases. Based on these, we attempted to mine the prognostic role of LARs, innovate LAR-based clinical subtypes and construct LAR-associated prognostic model to better understand the potential roles of LARs in further clinical applications.

In this study, we utilized RNA-seq data or RNA microarray data for 1,125 gliomas from the CGGA (*n* = 307), TCGA (*n* = 598) and GSE16011 (*n* = 250) datasets, and matched copy number variation (CNV; *n* = 598) and single nucleotide polymorphism (SNP; *n* = 583) data from the TCGA dataset. Based on bioinformatic and statistical analyses of these open-source datasets, several LARs were found to be involved in malignant progression and prognosis of glioma, and a predictive independent risk signature involving 14 screened LARs was developed to predict the prognosis of glioma patients. The results showed that several LARs were included in the frequent chromosome alterations observed in gliomas and show prognostic values. Tumor mutation burden (TMB) was also calculated for samples with mutation data in the TCGA dataset and we found TMB showed a positive correlation with our risk score, which may mean that DNA repair system is highly impaired in gliomas with higher risk score and dysregulation of lysine acetylation may lead to malignant progression in glioma. Overall, mRNA expressions and genome alterations of LARs were significant associated with the clinical outcomes of glioma patients and our study provides a method to perform clinical applications of them.

## Materials and Methods

### Data Acquisition

The RNA-seq data and corresponding clinicopathological information for the CGGA training set were downloaded from the CGGA website (http://www.cgga.org.cn/). The RNA-seq data, CNV data, and clinicopathological data for the validation set in TCGA were downloaded from the University of California, Santa Cruz Xena browser (UCSC Xena; https://xenabrowser.net/datapages/). SNP data in the TCGA dataset were downloaded from the Genomic Data Commons Data Portal (GDC; https://portal.gdc.cancer.gov/). The microarray mRNA expression profile of the GSE16011 dataset was downloaded from the Gene Expression Omnibus (GEO) repository (https://www.ncbi.nlm.nih.gov/geo/) and the corresponding clinical information was found in previous publication (Gravendeel et al., 2009). Immunohistochemistry images of LARs were obtained from the website of The Human Protein Atlas (HPA: https://www.proteinatlas.org/) The clinicopathological information for the CGGA and TCGA datasets is summarized in Supplementary Table S1. Copy number variation information of the 33 LARs is summarized in Supplementary Table S2.

### Data Processing

The RNA-seq transcriptome data for the CGGA and TCGA samples were normalized by log2 (*n*+1) transformation. For the GSE16011 microarray dataset, the raw data were downloaded to perform normalization processing using a robust multiarray averaging method (RAM) with the R packages “affy” (Gautier et al., 2004; Wilson and Miller 2005) and “simpleaffy” (Wilson and Miller 2005). The GISTIC2 method was applied to generate gene-level copy number estimates. GISTIC2 further thresholded the estimated values to −2, −1, 0, 1, 2, representing homozygous deletion, single copy deletion, diploid normal copy, low-level copy number amplification, or high-level copy number amplification, respectively.

### Selection of LARs

A list of LARs was compiled from the published literature and subsequently restricted to genes for which RNA expression data was available in both the CGGA and TCGA datasets. We obtained a final list of 33 LARs consisting of 13 lysine acetyltransferases and 20 lysine deacetylases. The extracted mRNA expression matrix of these 33 genes was used for the subsequent bioinformatics analysis.

Consensus cluster Consensus clustering and screening of molecular subtypes based on the expression profiles of the LARs were performed using the R package “ConsensusClusterPlus” (Wilkerson and Hayes 2010). The Euclidean distance was utilized to compute the similarity distance between samples, and the k-means method was used for clustering based on 50 iterations, with each iteration containing 80% of samples. Then principal component analysis (PCA) was performed to evaluate different expression patterns among glioma subgroups using the R programming language (https://www.r-project.org/).

### Functional Enrichment Analysis

Differential gene expression analysis between the LA3 and LA1/2 subgroups was performed using the R package “limma” (Ritchie et al., 2015), and the differentially expressed genes were input into the Database for Annotation, Visualization, and Integrated Discovery (DAVID) (https://david.ncifcrf.gov/) for GO and KEGG pathway enrichment analyses. GSEA software (http://software.broadinstitute.org/gsea/index.jsp) was used to investigate the enriched tumor hallmarks in the LA3 subgroup compared with those in the LA1/2 subgroups.

### Protein-Protein Interaction Network

Protein-protein interactions (PPI) among LARs were evaluated using the STRING database (https://string-db.org/), and the “Cytoscape” software was used to perform the visualization of the PPI network (Shannon et al., 2003).

### Calculation of the Tumor Mutation Burden

The tumor mutation burden (TMB) was calculated using Perl scripts (https://www.perl.org/ ), and the algorithm to calculate the TMB included nonsynonymous mutation counts per tumor, with germline mutations filtered out.

### Construction of the LAR-Related Prognostic Model

Based on the expression of 33 LARs in the CGGA dataset, univariate Cox regression analyses were used to judge their prognostic powers. We screened 23 genes associated with OS (*p* < 0.05) and used the LASSO Cox regression algorithm to develop a risk signature. Finally, 14 genes with their coefficients were determined according to minimum criteria, which involved selecting the best penalty parameter *λ* associated with the smallest 10-fold cross-validation within the training dataset. The risk score for the signature was calculated using the following formula:

$$\text{Risk score}=\sum_{i=1}^{n}{\text{Coef}}_{i}\text{*}x_{i}$$

in which Coef_*i*_ $Coef_{i}$ is the coefficient, and *x*_*i*_ $x_{i}\;$ is the log2 (*n*+1)-transformed relative expression value for each screened gene. The formula was used to compute a risk score for each patient in both the CGGA, TCGA and GSE16011 datasets. 

### Statistical Analyses

Differential LAR expression levels among WHO grades and CNV status, between different 1p/19q codeletion status and different IDH mutation status and differential TMB levels between low- and high-risk gliomas were compared by the Wilcox test. Chi-square tests were used to compare the distribution of gender, age, WHO grade, IDH mutation status, and 1p/19q codeletion status and CNV status of LARs among the three subgroups (clustered by consensus expression of LARs) and/or between low- and high-risk subgroups in gliomas.

The prognostic abilities of the risk score and other clinicopathological characteristics were evaluated by univariate and multivariate Cox regression analyses. The prediction efficiency of our risk signature, age, and WHO grade for 1/3/5-years survival was assessed by receiver operating characteristic (ROC) curves. Kaplan–Meier curves used to compare the OS for patients in different groups were tested by the log-rank test. Spearman correlation test was performed to analyze the correlation between TMB and risk score. All statistical analyses were performed using R v.3.6.1 (https://www.r-project.org/) and SPSS Statistics 25 (https://www.ibm.com/products/software).

## Results

### Correlation Between mRNA Expressions and Clinicopathological Features

**FIGURE 1:**
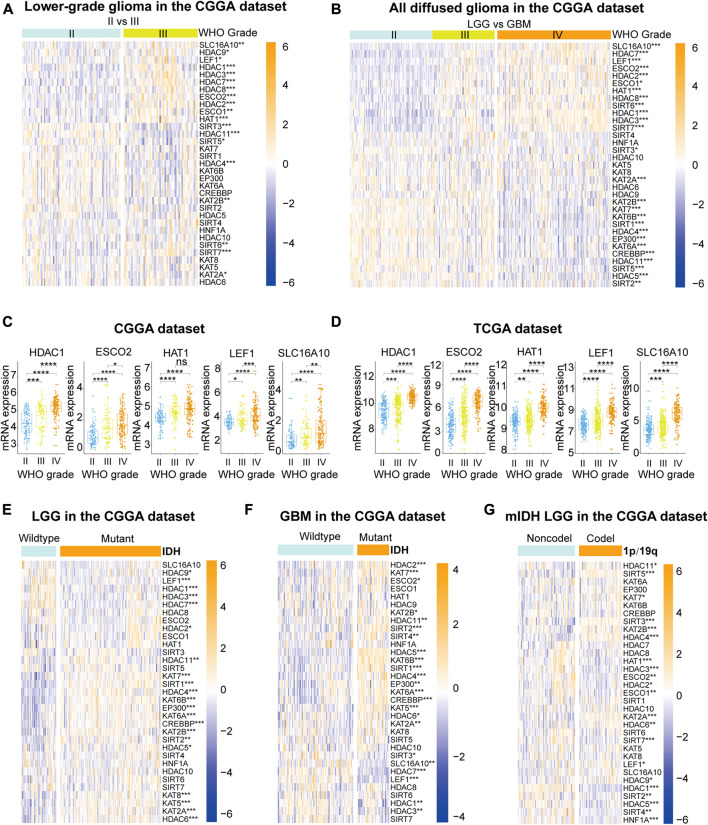
**(A)**, **(B)** The expression levels of 33 lysine acetylation regulators (LARs) in gliomas with different WHO grades. **(C)**, **(D)** HDAC1, ESCO2, KAT1, LEF1, and SCL16A10 expression levels increased with increasing WHO grade. **(E)**, **(F)** The expression levels of LARs in low-grade gliomas (LGGs) and glioblastomas (GBMs) with different IDH mutation status. **(G)** The expression levels of LARs in LGGs with IDH mutations (mIDH) with different 1p/19q codeletion status. (Wilcox test, *p < 0.05, **p < 0.01, ***p < 0.001, and ****p < 0.0001).

Given the crucial biological roles of each LAR, we systematically analyzed the correlation between LAR mRNA expression levels and clinicopathological characteristics (including WHO grades, IDH mutation status, and 1p/19q codeletion status) in gliomas. The heatmaps (Figures 1A,B) show the expression levels of each LAR in diverse WHO grades, and indicate that most of the LARs were aberrantly expressed in different WHO grades in the CGGA dataset; these differential expression levels were validated in the TCGA dataset (Supplementary Figures S1A,B). We found that the mRNA expression of 11 lysine acetyltransferases and 15 lysine deacetylases were significantly correlated with WHO grades in the CGGA dataset (Supplementary Figures S2A,B). For acetyltransferases, the mRNA expression levels of most KATs (except KAT5 and KAT8) decreased significantly with increasing WHO grade. In contrast, the mRNA expression of the other four acetyltransferases (SLC16A10, KAT1, ESCO1, and ESCO2) showed marked increases. For deacetylases, the mRNA expression of SIRT1/2/3/5, and that of HDAC4/5/11 decreased with increasing WHO grade, while the mRNA expressions of HDAC1/2/3/7/8, and that of SIRT6/7 and LEF1, showed an increase. Among these LARs that showed increased expression with increasing WHO grade in both the CGGA and TCGA datasets, HDAC1 is the best-studied LAR in glioma, whereas the potential functions of ESCO2, KAT1, LEF1, and SLC16A10 are unreported in this cancer (Figures 1C,D).

The differential expression levels of LARs according to IDH mutation status were investigated in LGGs and GBMs (Figures 1E,F). Our results showed that HDACs (except HDAC8 and 10), KATs (except KAT1), SIRT1, SIRT2, and LEF1 were all significantly associated with IDH mutation status in LGGs. The expression levels of HDAC1/2/3/4/5/6/7/10/11, as well as those of KATs (except KAT1 and KAT8), SIRT1/2/3/4, ESCO2, SLC16A10, and LEF1, were significantly correlated with IDH mutation status in GBMs. We also evaluated the mRNA expression of the 33 LARs according to 1p/19q codeletion status in LGGs with mutated IDH. We found that HDAC1/2/3/4/5/6/10/11, as well as KAT1/2A/2B/7, SIRT2/3/4/5/7, LEF1, TCF1, ESCO1/2 were closely associated with 1p/19q codeletion status in LGGs with mutated IDH (Figure 1G).

For further investigating the functional status of LARs in gliomas, we downloaded immunohistochemistry images of several LARs from the Human Protein Atlas database (Supplementary Figure S3). Most of investigated LARs (including HDAC1/2/3/5/8, SIRT5/7, KAT2A/2B and LEF1) were differential expressed between LGG and GBM, and have similar expression tendency with mRNA expression in our analysis.

### Identification of Glioma Subgroups by Consensus Clustering

**FIGURE 2:**
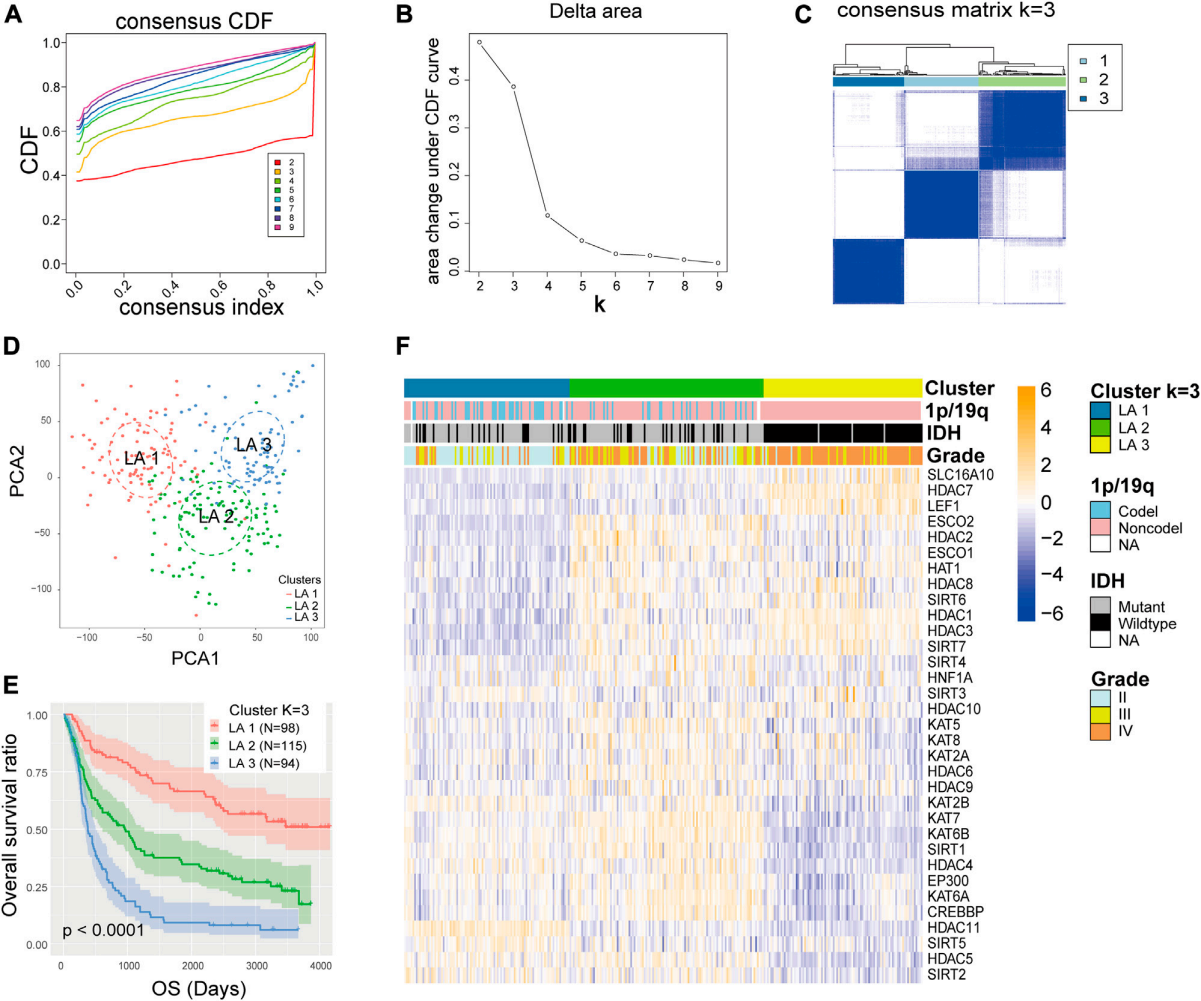
**(A)** Consensus clustering cumulative distribution function (CDF) for k = 2 to 9. **(B)** Relative change in area under the CDF curve for k = 2 to 9. **(C)** Consensus clustering matrix for k = 3. **(D)** Principal component analysis of the total RNA expression profile in the CGGA dataset. **(E)** Kaplan–Meier overall survival curves for 307 glioma patients in the LA1, 2, and three subgroups. **(F)** The different expression levels of lysine acetylation regulators (LARs) and clinicopathological feature contributions of the three subgroups defined by the consensus expression of 33 LARs.

The mRNA expression of the 33 LARs was analyzed to determine the glioma subtypes in the CGGA dataset. A total of 307 samples were divided into k (k = 2–9) subtypes using the R package “Consensus Cluster Plus”. We elected k = 3 (Figures 2A–C) as our subtype-dividing value for further study due to the similar number of samples in each cluster and distinct clinical prognoses among the subgroups when we divided gliomas into three subgroups. To investigate the differences among the three subgroups in more detail, we performed PCA to compare the mRNA expression profiles among the three subgroups and the analysis showed that significant differences existed among the three subgroups (Figure 2D). Furthermore, survival analysis was conducted and results showed that the LA3 subgroup had the poorest OS time and rate while the LA1 subgroup showed the longest OS time among the three groups (Figure 2E). Subsequently, we evaluated the differences in clinicopathological features and expression levels among the three clusters (LA1, LA2, and LA3) (Figure 2F), and found that, compared with the other two groups, LA3 was significantly related to increased age at diagnosis (*p* < 0.001), higher WHO grade (*p* < 0.001), fewer IDH mutations (*p* < 0.001), and fewer 1p/19q codeletions (*p* < 0.001) (Supplementary Table S3). In contrast, the other two subgroups correlated with younger age at diagnosis, lower WHO grade, more IDH mutations, and more 1p/19q codeletions.

### Gene Ontology and Gene Set Enrichment Analysis

**FIGURE 3:**
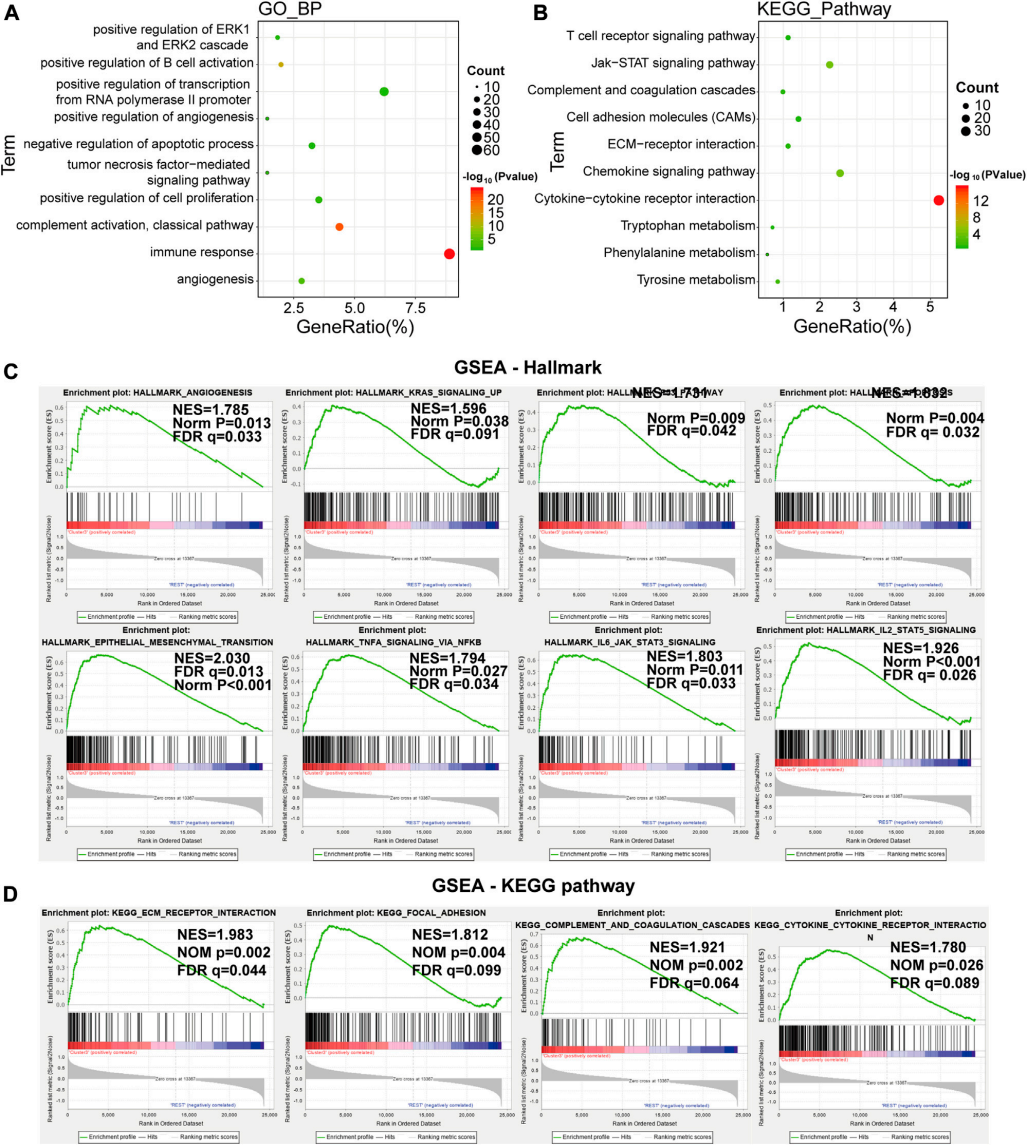
**(A)**, **(B)** Functional annotation of the genes with higher expression in the LA3 subgroup using gene ontology (GO) terms of biological processes **(A)** and Kyoto Encyclopedia of Genes and Genomes (KEGG) pathway **(B)** analyses. **(C)** Gene set enrichment analysis indicated that genes with higher expression in the LA3 subgroup were enriched for hallmarks of malignant tumors.

The above findings implied that clustering was closely related to glioma malignancy. As the LA3 subgroup had the poorest prognosis, we identified genes that were significantly upregulated (log(fold change) > 1 and *p* < 0.05) in the LA3 subgroup compared with the LA1 and LA2 subgroups, and annotated their functions by gene ontology (GO) pathway analysis for biological processes (BPs) and Kyoto Encyclopedia of Genes and Genomes (KEGG) analysis. The GO–BP results showed that the upregulated genes were enriched in malignancy-related biological processes, such as positive regulation of the ERK1 and ERK2 cascade, angiogenesis, cell proliferation, tumor necrosis factor-mediated signaling pathway, immune response, and negative regulation of apoptotic process (Figure 3A). Similar results, such as JAK–STAT signaling pathway, cell adhesion molecules (CAMs), and extracellular matrix (ECM)-receptor interaction, were also significantly enriched in the LA3 subgroup based on KEGG pathway analysis (Figure 3B). We also performed gene set enrichment analysis (GSEA) between the LA3 and LA1/2 subgroups. The results revealed that malignant hallmarks of tumors, including IL2/STAT5 signaling, epithelial–mesenchymal transition, apoptosis, p53 pathway, IL6/JAK/STAT3 signaling, angiogenesis, TNF signaling via NF-κB, and KRAS signaling, were enriched in the LA3 subgroup (Figure 3C). The GSEA-based KEGG pathway analysis verified that ECM receptor interaction, Focal adhesion, Complement and coagulation cascades and Cytokine-cytokine receptor interaction were enriched in LA3 subgroup (Figure 3D).

### Correlations and Interactions Among LARs

**FIGURE 4:**
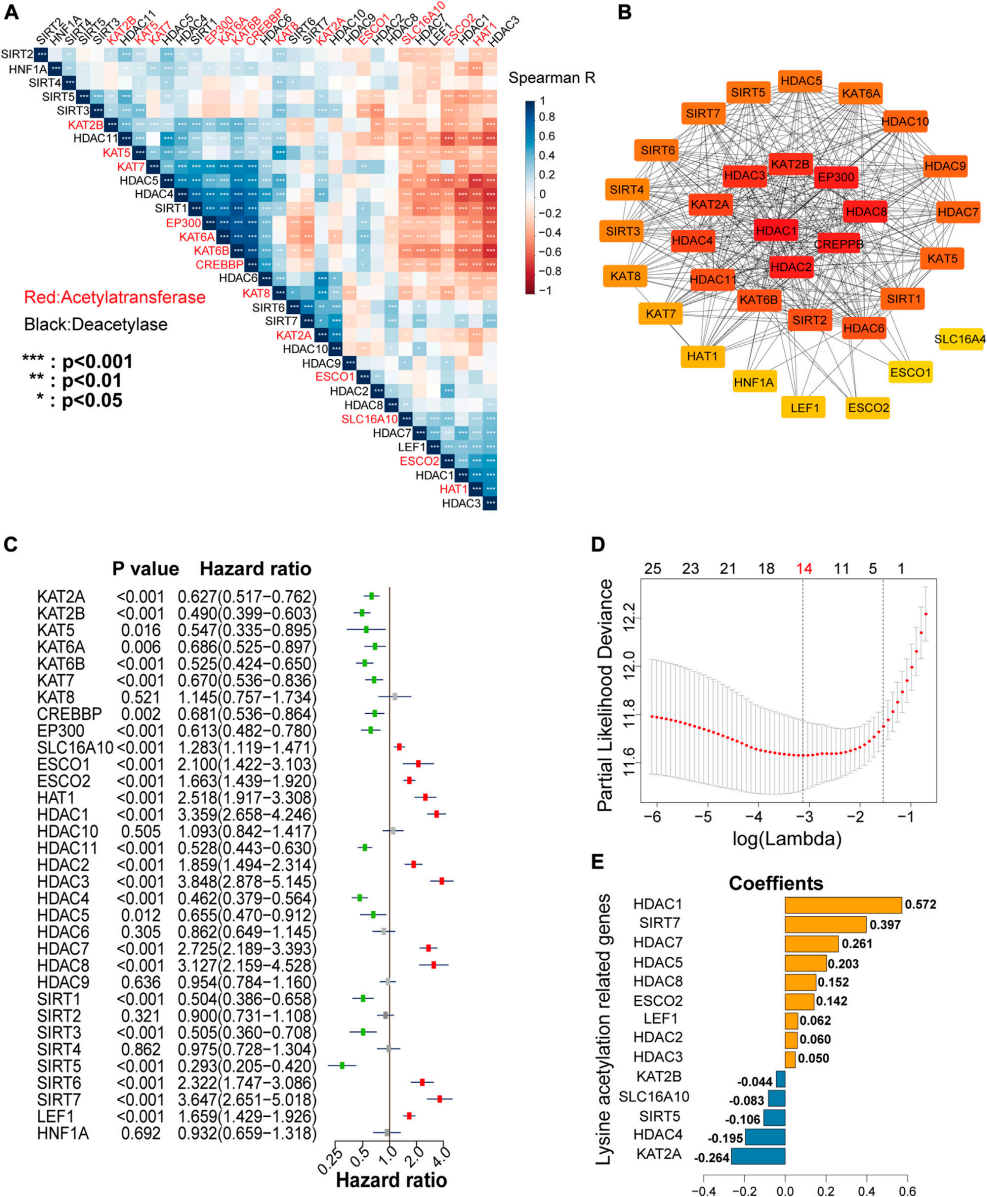
**(A)** Spearman’s correlation analysis among the 33 lysine acetylation regulators (LARs). **(B)** Protein interaction network of the 33 LARs. **(C)** Univariate Cox regression analysis was used to calculate the hazard ratios (HRs), 95% confidence intervals, and *p*-values for screening the prognostic LARs. **(D)**, **(E)** Least absolute shrinkage and selection operator (LASSO) regression was performed to calculate the minimum criteria **(D)** and coefficients **(E)**.

To better understand the correlations among the LARs, we performed correlative expression analysis and protein-protein interaction (PPI) network analyses. We found that genes within the same functional class showed significantly correlated expression patterns and that a high correlation existed between acetyltransferases and deacetylases (Figure 4A). In the correlation analysis, five acetyltransferases (KAT2B, KAT5, KAT6A, KAT6B, KAT7, EP300, and CREBBP) presented a strong co-expression relationship, they were also positively associated with the expression of HDAC4, HDAC5, HDAC6, and SIRT1 and negatively associated with SLC16A10, HAT1, HDAC1, HDAC3, and LEF1 expression. The HDAC family seems to be the hub family in lysine regulation, as it showed strong co-expression not only among the family members, but also with that of KATs and SIRTs. In contrast, few SIRTs (except SIRT1) showed strong correlations with the other LARs.

Analysis of PPI networks also showed that these LARs frequently interacted (Figure 4B), and that HDAC1, HDAC2, and CREBBP presented the greatest number of links to other LARs. In the PPI networks, we concluded that HDACs had an especially high number of interactions with other LARs compared with other lysine acetylation regulator family. Taken together, these findings revealed that several co-expression patterns existed among the LARs, and HDACs are the hub family.

### Building a Risk Signature by LASSO Cox Regression

To investigate the prognostic value of LARs, univariate Cox regression analysis was performed on the mRNA expression of the 33 LARs in the CGGA training dataset. We found that 26 of the 33 genes were correlated with OS (*p* < 0.05) of glioma patients (Figure 4C). Among the 26 genes, SLC16A10, ESCO1, ESCO2, KAT1, HDAC1, HDAC2, HDAC3, HDAC7, HDAC8, SIRT6, SIRT7, and LEF1 were found to be risk factors in glioma, with hazard ratios >1, whereas KAT2A, KAT2B, KAT5, KAT6A, KAT6B, KAT7, CREBBP, EP300, HDAC11, HDAC4, HDAC5, SIRT1, SIRT3, and SIRT5 were protective factors, with hazard ratios <1. The 26 LARs identified as having prognostic value were selected for use with the least absolute shrinkage and selection operator (LASSO) Cox regression algorithm in the CGGA training dataset. Based on the minimum criteria, we determined a 14-gene risk signature (Figure 4D), and the coefficients (Figure 4E) obtained by the LASSO algorithm were used to compute the risk score for each sample in the CGGA and TCGA datasets for further study. Besides, prognostic value of each LARs in LGG and GBM with different IDH mutant status and 1p/19q codeletion status were concluded in Supplementary Tables S4 and S5.

### Testing and Validating the Risk Signature

**FIGURE 5:**
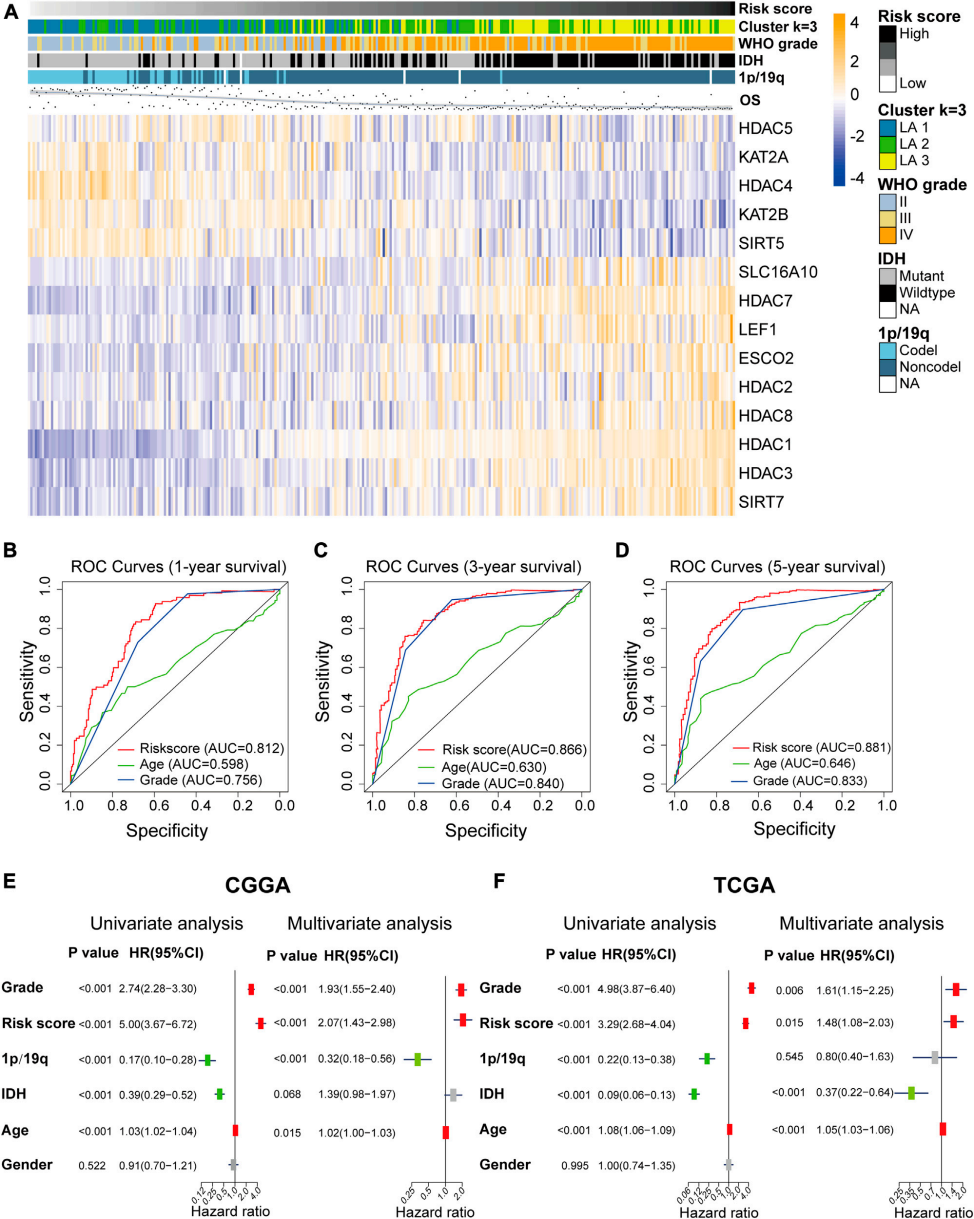
**(A)** The differential expression levels of the included 14 lysine acetylation regulators (LARs) and the distributions of clinicopathological characteristics were compared between low- and high-risk subgroups. **(B)**–**(D)** Receiver operating characteristic (ROC) curves showed the predictive efficiency of the risk signature, WHO grade, and age on 1/3/5-years survival rate. **(E)**, **(F)** Univariate and multivariate Cox regression analyses of the overall survival and clinicopathological features of patients from the Chinese Glioma Genome Atlas (CGGA) **(E)** and The *Cancer* Genome Atlas (TCGA) **(F)** datasets.

We plotted heatmaps to evaluate whether the risk score reflected the different distributions of clinicopathological features among gliomas in the CGGA dataset (Figure 5A) and the TCGA dataset (Supplementary Figure S4A). Significant differences in clinicopathological features were observed between the low- and high-risk subgroups (Supplementary Table S6). The high-risk subgroup was highly associated with older age, higher WHO grade, wild-type IDH, and non-codeletion of 1p/19q, and OS time decreased with the risk score increasing. Based on the ROC curves, we concluded that the risk score could perfectly predict 1/3/5-years survival rates in glioma patients with AUC = 0.812/0.866/0.881, respectively (Figures 5B–D), and was more efficient than WHO grade and age. ROC curves in the TCGA validation set proved that the risk signature had a stable and robust predictive ability (Supplementary Figures S4B–D).

Univariate and multivariate Cox regression analyses were performed to determine whether the risk signature was an independent prognostic indicator. We included age, risk score, 1p/19q codeletion status, WHO grade, IDH mutation status, gender in the univariate Cox regression analysis and the results of the univariate and multivariate Cox regression analysis showed that risk score, WHO grade, age and 1p/19q status were independent predictors for glioma patients (Figures 5E,F).

**FIGURE 6:**
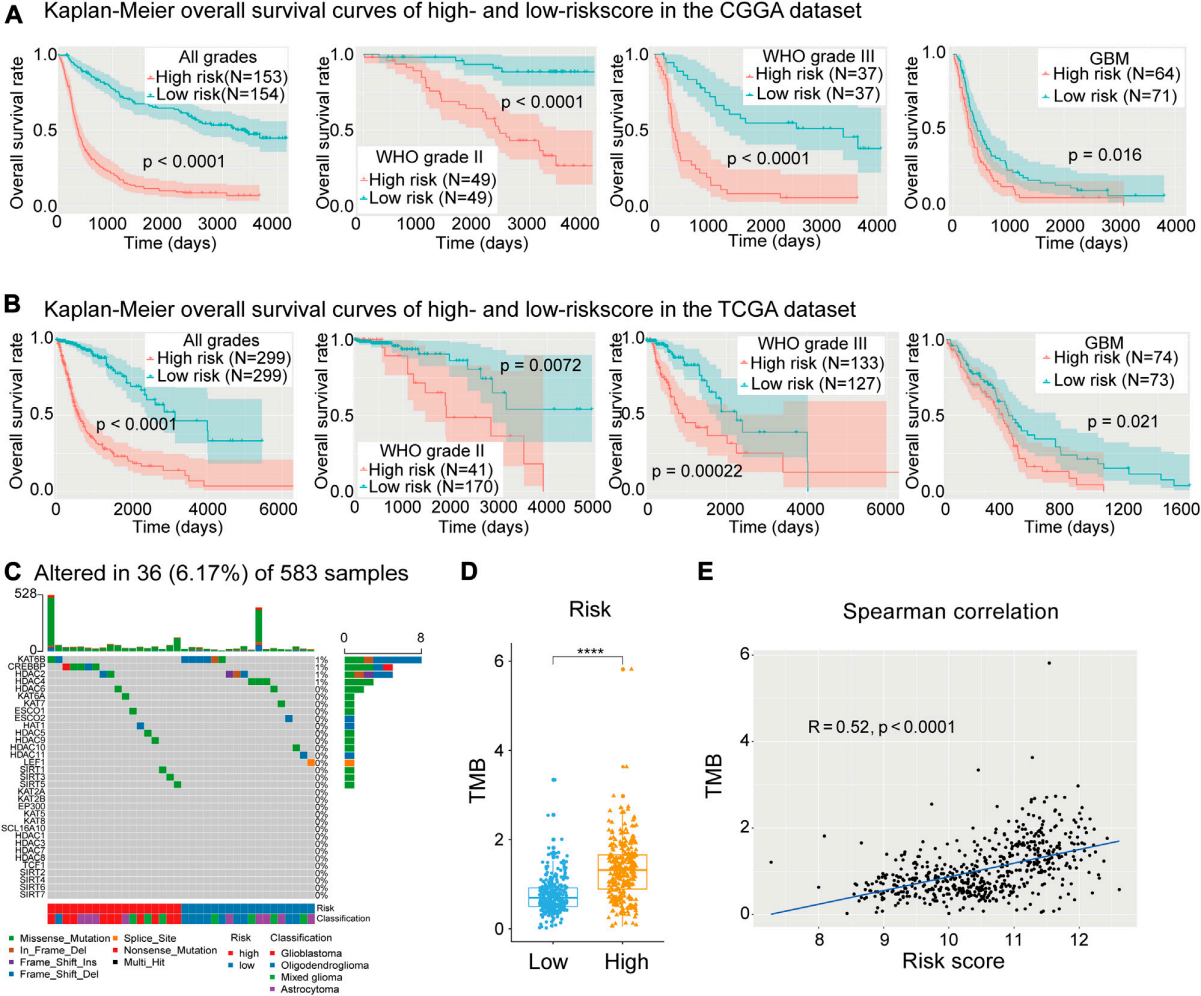
**(A)** Kaplan–Meier curves of low- and high-risk subgroups of all grades and each grade in the Chinese Glioma Genome Atlas (CGGA) training dataset. **(B)** Kaplan–Meier curves of low- and high-risk subgroups of each grade in The *Cancer* Genome Atlas (TGGA) training dataset. **(C)** Waterfall plot depicting the mutant status of lysine acetylation regulators (LARs). **(D)**, **(E)** Box plot showing that risk subgroups were significantly associated with the tumor mutation burden (TMB) and Spearman correlation analysis showed that the risk score was strongly correlated with TMB. (Wilcox test, *p < 0.05, **p < 0.01, ***p < 0.001, and ****p < 0.0001).

Here, we confirmed that the risk score had prognostic value for different WHO grades. The Kaplan–Meier survival curves indicated that low-risk patients had longer OS time and a higher OS rate than high-risk patients in each and all WHO grades in the CGGA dataset (Figure 6A), and the prognostic ability of the risk score was further validated in the TCGA dataset (Figure 6B).

### Further Validation in the GSE16011 Microarray Dataset

On account of the expression file of TCGA and CGGA sets were both RNA-seq data, we applied the risk score model in the GSE16011 microarray dataset to evaluate the universality of the LAR-signature. Similarly, in the GSE16011 dataset, patients with higher risk score showed shorter OS time and rate in all grade gliomas, LGGs and GBMs (Supplementary Figures S5A–C). The prognosis predictive ability of the risk score was also compared with the WHO grade and age of patients in the GSE16011 dataset by using the model of ROC curves. The risk score obtained high AUC values (0.703/0.765/0.754) for predicting 1/3/5-years OS time. Particularly, the AUC values of risk score were higher than age and WHO grade of patients for predicting 3/5-years OS time (Supplementary Figures S5D–F). Besides, univariate and multivariate Cox regression analysis showed the risk score was an independent predictive factor in the GSE16011 microarray dataset (Supplementary Figures S5G,H).

### Mutation Analysis of LARs

To study the mutation status of the 33 LARs and the relationship between the risk signature and gene mutation load, 583 samples with matched SNP data were divided into low- (n = 303) and high-risk (n = 280) groups. A waterfall plot was generated depicting the mutation frequency of the 33 LARs and the percentage at which they occurred in gliomas (Figure 6C). We found that 36 (6.17%) of the 583 samples contained mutations in genes coding for the LARs, in which KAT6B (8/583) and CREBBP (5/583) were the most frequently mutated genes. Within the eight mutations found in KAT6B, six were present in oligodendrogliomas and six of the samples were in the low-risk subgroup. All the mutations in CREBBP were in samples from high-risk patients, and comprised two glioblastomas and three astrocytomas.

In the waterfall plot depicting the 30 most frequently mutated genes in gliomas (Supplementary Figure S6A), we noticed that glioma patients with a high-risk score often carried a higher frequency of gene mutations. This indicated that DNA repair system is highly damaged in patients with higher risk score. Therefore, we calculated the TMB for each sample with SNP data in the TCGA dataset. We found that high-risk patients had higher TMB values (Figure 6D), and Spearman’s correlation analysis confirmed the positive correlation between our risk signature and TMB (Figure 6E). This result implied that impaired regulation of lysine acetylation may affect glioma malignancy through the modulation of factors involved in DNA replication or repair.

### CNV Analysis of LARs

**FIGURE 7:**
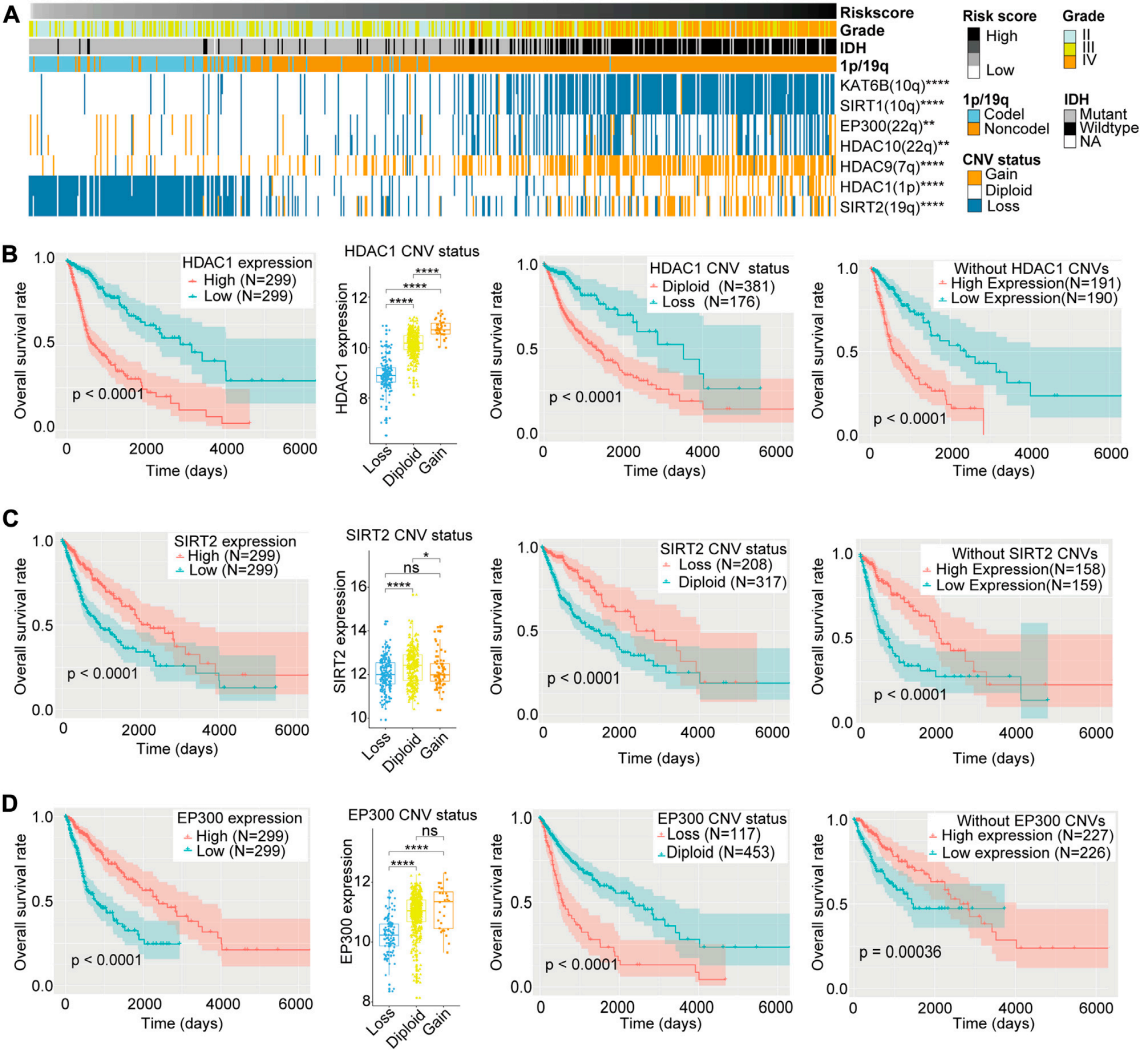
**(A)** Copy number variation (CNV) of seven lysine acetylation regulators (LARs) were significantly correlated with the risk score and other clinicopathological features (Chi-square tests). **(B)**–**(D)** Box plots indicated that different CNV status of HDAC1, SIRT2, and EP300 have different mRNA expression levels and the Kaplan–Meier curves revealed that HDAC1, SIRT2, and EP300 expression levels were associated with OS rates both in all samples and samples without their CNVs and their CNV status were also related to OS rates of glioma patients. (Wilcox test, *p < 0.05, **p < 0.01, ***p < 0.001, and ****p < 0.0001).

In the heatmap depicting the CNV of the 33 LARs, the high-risk section of the heatmap showed more CNVs than the low-risk section (Supplementary Figure S6B). We selected seven LARs for which the CNV was highly associated with the risk score for further analyses (Figure 7A). These genes are located on chromosome arms 1p, 7p, 10q, 19q, and 22q which are characteristically altered in gliomas (Bredel et al., 2009).

HDAC1 and SIRT2 are located on chromosome arms 1p and 19q, respectively. Codeletion of these genes is frequently observed in oligodendrogliomas, and is highly associated with improved responses to radiochemotherapy and longer survival than diffuse gliomas without these alterations (Cancer Genome Atlas Research Network et al., 2015). Although Kaplan–Meier curves revealed that copy number deletions of HDAC1 and SIRT2 are related to a better prognosis, we could not determine whether 1p/19q codeletions resulted in differential OS. Therefore, to exclude the potential influences of 1p/19q codeletion, we compared the mRNA expression levels of HDAC1 and SIRT2 according to CNV status, as well as the OS rates between low and high levels of HDAC1 and SIRT2 mRNA expression in gliomas without HDAC1 or SIRT2 CNVs (Figures 7B,C). We found that, for both genes, copy number deletions were associated with lower mRNA expression, and in gliomas without HDAC1 or SIRT2 copy number variations, patients with lower HDAC1 expression or higher SIRT2 expression showed better clinical prognosis. These results indicated that HDAC1 may be one of the oncogenes lost in gliomas with 1p deletion, while SIRT2, as a protective factor, is lost with 19q codeletions in glioma patients.

The EP300 is located on chromosome 22q, and deletion of this gene is also common in gliomas. Although EP300 copy number deletion was associated with worse prognosis when compared with the diploid state, we could not exclude that loss of other genes located in 22q may also influence prognosis. Therefore, we compared EP300 mRNA expression levels according to CNV status, as well as the OS rates between high and low levels of EP300 expression in gliomas without EP300 CNVs. We found that copy number deletions of EP300 were associated with lower EP300 mRNA expression levels, and reduced expression of EP300 in gliomas without EP300 CNVs was related to a worse clinical prognosis (Figure 7D). This indicates that EP300 may play a tumor suppressor role in glioma and EP300 (22q) may be one of the tumor suppressor genes lost in the 22q− event (Hartmann et al., 2004).

The CNVs for the other four LARs–KAT6B (10q), SIRT1(10q), HDAC10 (22q), and HDAC9 (7q)–were highly associated with the risk signature and may be affected in chromosomal alterations such as 10q−, 22q− and 7+ in gliomas. However, we did not find significant differences in OS between low and high levels of expression of these four genes in patients without CNVs (Supplementary Figures S7A–D). Therefore, we regarded the differences in OS rates between patients with or without copy number loss of these four genes as passive changes resulting from chromosomal variations, indicating that they may have little effect in related clinical outcomes.

## Discussion

In this report, we have shown that the mRNA expression levels of most of the evaluated LARs are closely associated with clinicopathological features of glioma. We further identified three subgroups (LA1/2/3) of gliomas by consensus clustering of 26 OS-related LARs, and confirmed that LA3 was the most malignant subtype with the poorest prognosis. Moreover, the LA3 subgroup was tightly associated with malignancy-related biological processes, key signaling pathways, and tumor hallmarks. In addition, we also constructed a prognostic signature and divided glioma patients into low- and high-risk categories by the median of risk scores. We noticed a close relationship between the risk signature and clinicopathological features of glioma, and ROC curves, univariate and multivariate analyses, and Kaplan–Meier curves were used to determine the potential prognosis value of the risk signature in glioma. We also included SNP and CNV data of LARs to identify potential therapeutic targets that may play a prognostic role in gliomas.

The advantages of our study included that we analyzed comprehensive genome and transcriptome data of 1,125 glioma patients and we build a LAR-based glioma classification system and LAR-related risk score model which was validated in two external independent glioma cohorts. The LAR-signature possessed strong and steady prognostic value and it’s promising in further clinical application. With the rapid evolution of bioscience and bioinformatics, it’s convenient in using sequencing technique for clinical diagnosis and prognostic assessment. How to combine computational technique with bioscience tools in clinical use would break through the difficulties in patient diagnosis and prognosis prediction. Thus, we thought our work could provide a strong weapon for predicting the prognosis of glioma patients. But the limitations were also obvious, the LAR-signature need more perspective rather than retrospective validations for further clinical using. Besides, we thought the underlying functions of LARs also need experimental verifications in following research.

We have found that the LAR-signature was pertinent to the TMB of glioma patients. The TMB is associated with neoantigen abundance and increased immunogenicity (Hodges et al., 2017), and is used to quantitatively assess mutations carried by tumor cells, which was usually used to predict the response to immunotherapy in cancer patients It is defined as the total number of somatic gene coding errors, base substitutions, gene insertions, or deletion errors that are detected per million bases. In recent years, several studies have demonstrated that dysregulation of lysine acetylation may result in errors during DNA damage repair. For instance, PCAF/GCN5-mediated K163 acetylation of RPA1 (replication protein A) is crucial for nucleotide excision repair (NER) (Zhao et al., 2017), SIRT7 is recruited in a PARP1-dependent manner to sites of DNA damage, where it modulates H3K18Ac levels (Vazquez et al., 2016), and TET1 (ten-eleven translocation-1) forms a complex with KAT8 to modulate its function and the level of H4K16Ac, which ultimately affects gene expression and DNA repair (Zhong et al., 2017). Based on these observations, we speculate that dysregulation of lysine acetylation of both histone and non-histone proteins may play a pivotal role in impairing the DNA damage repair response, which would then lead to hypermutations and an increased neoantigen load, leading to malignant progression of tumors. We have systematically revealed the mRNA expression, underlying functions, and prognostic values of LARs in glioma, and shown that acetylation regulators may have an immune-related effect on the malignant progression of glioma. Moreover, we identified that several underexplored LARs, such as *ESC O 2*, *HAT1(KAT1)*, and *LEF1*, may have prognostic value in lower grade glioma patients (Supplementary Table S5) and may be potential glioma biomarkers. We further found that specific chromosomal alterations in gliomas were highly related to the CNVs of several LARs. *HDAC1* was shown to be one of the oncogenes deleted in the 1p deletion event, and *SIRT2* and *EP300* were two cancer suppressors lost in 19q deletion and 22q deletion events, respectively. Our results also revealed that dysfunction of LARs may partially explain the hypermutation state of gliomas, which is associated with unfavorable prognosis. Besides, which is the main idea of our research, LAR-based glioma subtypes were built and LAR-associated risk scores were constructed to expand the potential clinical applications of LARs. Taken together, we believe that substantial research is still required to illuminate the detailed mechanisms involved in lysine acetylation-mediated regulation of glioma malignancy that may ultimately lead to new and effective targeted therapies for glioma patients, and the use of LAR-subtype and LAR-signature are promising for further clinical applications and need more perspective evidence.

## Data Availability

Publicly available datasets were analyzed in this study. These data can be found here: the Chinese Glioma Genome Atlas (CGGA)(http://www.cgga.org.cn/), the University of California, Santa Cruz Xena browser (UCSC Xena; https://xenabrowser.net/datapages/), the Genomic Data Commons Data Portal (GDC; https://portal.gdc.cancer.gov/), the Gene Expression Omnibus (GEO) repository https://www.ncbi.nlm.nih.gov/geo/) and The Human Protein Atlas (HPA: https://www.proteinatlas.org/).
