# Supplementary material for: Systematic and Multi-Omics Prognostic Analysis of Lysine Acetylation Regulators in Glioma
Source: Front Mol Biosci. 2021 Feb 26;8:587516. doi: 10.3389/fmolb.2021.587516 (PMC7954118; doi:10.3389/fmolb.2021.587516)
Supplement: Supplementary file 1 [file DataSheet1.docx]

**Figure S1. (A, B)** The expression levels of 33 lysine acetylation regulators (LARs) in gliomas with different WHO grades in the TCGA dataset. **(C)** The expression levels of 33 lysine acetylation regulators (LARs) in LGGs with different IDH status in the TCGA dataset. **(D)** The expression levels of 33 lysine acetylation regulators (LARs) in LGGs with different 1p/19q status in the TCGA dataset.

**Figure S2. (A)** Lysine acetyltransferase mRNA expression levels among WHO grades in the CGGA dataset. **(B)** Lysine deacetylase mRNA expression levels among WHO grades in the CGGA dataset.

**Figure S3**. **(A-J)** Immunohistochemistry images of 10 LARs were obtained from the HPA (The Human Protein Atlas) dataset.

**Figure S4. (A)** The expression levels of the included 14 lysine acetylation regulators (LARs) and the distributions of clinicopathological characteristics were ordered by the risk scores. **(B-D)** Receiver operating characteristic (ROC) curves showed the predictive efficiency of the risk signature, WHO grade, and age on 1/3/5-year survival rate in the TCGA dataset.

**Figure S5. (A-C)** The survival analysis between low- and high-risk gliomas in all grades or subtype gliomas in the GSE16011 dataset. **(D-F)** ROC curves of risk score, age and WHO grade in predicting the 1/3/5-year OS of glioma patients in the GSE16011 dataset. **(G-H)** Univariate and multivariate Cox regression analysis of factors including age, gender, WHO grade and risk score in the GSE16011 dataset.

**Figure S6. (A)** The mutation landscape of low- and high-risk gliomas in the TCGA dataset. **(B)** Copy number variations of 33 LARs in the gliomas ordered by the risk scores in the TCGA dataset.

**Figure S7. (A-D)** Box plots depicted that the mRNA expression levels of different HDAC9, HDAC10, KAT6B and SIRT1 CNV status and the Kaplan–Meier curves revealed that HDAC9, HDAC10, KAT6B and SIRT1 expression levels were not associated with OS rates in samples without their CNVs.
