# Supplementary material for: Systematic and Multi-Omics Prognostic Analysis of Lysine Acetylation Regulators in Glioma
Source: Front Mol Biosci. 2021 Feb 26;8:587516. doi: 10.3389/fmolb.2021.587516 (PMC7954118; doi:10.3389/fmolb.2021.587516)
Supplement: Supplementary file 2 [file Table1.docx]

| **Supplemental Table 1. Clinicopathological features of glioma patients included in this study** | | | | | |
| --- | --- | --- | --- | --- | --- |
|  |  | **CGGA dataset** | | **TCGA dataset** | |
|  |  | **Number** | **Percentage** | **Number** | **Percentage** |
| **Total** |  | 307 | 100.00% | 598 | 100.00% |
| **Age** |  |  |  |  |  |
|  | <median | 142 | 46.25% | 295 | 49.33% |
|  | ≥median | 165 | 53.75% | 303 | 50.67% |
| **Gender** |  |  |  |  |  |
|  | Male | 190 | 61.89% | 349 | 58.36% |
|  | Female | 117 | 38.11% | 249 | 41.64% |
| **WHO grade** |  |  |  |  |  |
|  | II | 98 | 31.92% | 211 | 35.28% |
|  | III | 74 | 24.10% | 240 | 40.13% |
|  | IV | 135 | 43.97% | 147 | 24.58% |
| **IDH** |  |  |  |  |  |
|  | Mutant | 167 | 54.40% | 372 | 62.21% |
|  | Wildtype | 139 | 45.28% | 220 | 36.79% |
|  | NA | 1 | 0.33% | 6 | 1.00% |
| **1p/19q** |  |  |  |  |  |
|  | Codel | 63 | 20.52% | 149 | 24.92% |
|  | Non-codel | 239 | 77.85% | 449 | 75.08% |
|  | NA | 5 | 1.63% | 0 | 0.00% |

| **Supplemental Table 2. Different CNV patterns occur in TCGA glioma samples (n=598)** | | | | | | | | | | |
| --- | --- | --- | --- | --- | --- | --- | --- | --- | --- | --- |
|  | **Gene** | **Diploid** | **Deep deletion** | **Shallow deletion** | **Loss sum** | **Copy number gain** | **Amplification** | **Gain sum** | **CNV sum** | **Percentage (CNV/Total)** |
| **Acetylatransferase** | CREBBP | 529 | 0 | 36 | 36 | 29 | 4 | 33 | 69 | 11.54% |
|  | EP300 | 453 | 4 | 113 | 117 | 27 | 1 | 28 | 145 | 24.25% |
|  | ESCO1 | 494 | 1 | 70 | 71 | 31 | 2 | 33 | 104 | 17.39% |
|  | ESCO2 | 514 | 0 | 32 | 32 | 51 | 1 | 52 | 84 | 14.05% |
|  | HAT1 | 548 | 1 | 28 | 29 | 21 | 0 | 21 | 50 | 8.36% |
|  | KAT2A | 527 | 2 | 35 | 37 | 34 | 0 | 34 | 71 | 11.87% |
|  | KAT2B | 526 | 1 | 36 | 37 | 34 | 1 | 35 | 72 | 12.04% |
|  | KAT5 | 506 | 0 | 44 | 44 | 46 | 2 | 48 | 92 | 15.38% |
|  | KAT6A | 514 | 0 | 27 | 27 | 55 | 2 | 57 | 84 | 14.05% |
|  | KAT6B | 371 | 1 | 222 | 223 | 4 | 0 | 4 | 227 | 37.96% |
|  | KAT7 | 529 | 0 | 29 | 29 | 40 | 0 | 40 | 69 | 11.54% |
|  | KAT8 | 534 | 0 | 40 | 40 | 21 | 3 | 24 | 64 | 10.70% |
|  | SLC16A10 | 482 | 0 | 106 | 106 | 10 | 0 | 10 | 116 | 19.40% |
| **Deacetylase** | HDAC1 | 381 | 1 | 175 | 176 | 41 | 0 | 41 | 217 | 36.29% |
|  | HDAC2 | 475 | 6 | 107 | 113 | 10 | 0 | 10 | 123 | 20.57% |
|  | HDAC3 | 518 | 3 | 60 | 63 | 16 | 1 | 17 | 80 | 13.38% |
|  | HDAC4 | 513 | 21 | 48 | 69 | 16 | 0 | 16 | 85 | 14.21% |
|  | HDAC5 | 532 | 1 | 31 | 32 | 34 | 0 | 34 | 66 | 11.04% |
|  | HDAC6 | 455 | 5 | 116 | 121 | 20 | 2 | 22 | 143 | 23.91% |
|  | HDAC7 | 497 | 7 | 68 | 75 | 26 | 0 | 26 | 101 | 16.89% |
|  | HDAC8 | 450 | 1 | 90 | 91 | 49 | 8 | 57 | 148 | 24.75% |
|  | HDAC9 | 370 | 0 | 10 | 10 | 214 | 4 | 218 | 228 | 38.13% |
|  | HDAC10 | 443 | 6 | 123 | 129 | 25 | 1 | 26 | 155 | 25.92% |
|  | HDAC11 | 523 | 1 | 35 | 36 | 38 | 1 | 39 | 75 | 12.54% |
|  | SIRT1 | 373 | 1 | 220 | 221 | 4 | 0 | 4 | 225 | 37.63% |
|  | SIRT2 | 317 | 5 | 203 | 208 | 67 | 6 | 73 | 281 | 46.99% |
|  | SIRT3 | 443 | 21 | 102 | 123 | 30 | 2 | 32 | 155 | 25.92% |
|  | SIRT4 | 503 | 4 | 59 | 63 | 25 | 7 | 32 | 95 | 15.89% |
|  | SIRT5 | 518 | 5 | 61 | 66 | 12 | 2 | 14 | 80 | 13.38% |
|  | SIRT6 | 413 | 1 | 31 | 32 | 139 | 14 | 153 | 185 | 30.94% |
|  | SIRT7 | 502 | 1 | 34 | 35 | 51 | 10 | 61 | 96 | 16.05% |
|  | LEF1 | 447 | 2 | 107 | 109 | 10 | 2 | 12 | 121 | 21.30% |
|  | HNF1A | 504 | 3 | 59 | 62 | 25 | 7 | 32 | 94 | 15.72% |

| **Supplemental Table 3. Clinicopathological features are different among LA1/2/3 subgroups** | | | | | |
| --- | --- | --- | --- | --- | --- |
|  |  | **Cluster1** | **Cluster2** | **Cluster3** | **P-value** |
| **Total case** | | **98** | **115** | **94** |  |
| **Gender** |  |  |  |  | 0.768 |
|  | Male | 59 | 70 | 61 |  |
|  | Female | 39 | 45 | 33 |  |
| **Age** |  |  |  |  | <0.001 |
|  | >42 | 48 | 41 | 76 |  |
|  | <42 | 50 | 74 | 18 |  |
| **WHO grade** | |  |  |  | <0.001 |
|  | II | 66 | 29 | 3 |  |
|  | III | 18 | 40 | 16 |  |
|  | IV | 14 | 46 | 75 |  |
| **IDH** |  |  |  |  | <0.001 |
|  | Mutant | 76 | 88 | 3 |  |
|  | Wildtype | 21 | 27 | 91 |  |
|  | NA | 1 | 0 | 0 |  |
| **1p/19q** |  |  |  |  | <0.001 |
|  | Codeletion | 40 | 23 | 0 |  |
|  | Non-codeletion | 56 | 90 | 93 |  |
|  | NA | 2 | 2 | 1 |  |

| **Supplemental Table 4. Clinicopathological features are different between low- and high-risk subgroups** | | | | |
| --- | --- | --- | --- | --- |
|  |  | **Low-risk** | **High-risk** | **P-value** |
| **Total case** |  | **154** | **153** |  |
| **Gender** |  |  |  | 0.311 |
|  | Male | 91 | 99 |  |
|  | Female | 63 | 54 |  |
| **Age** |  |  |  | 0.014 |
|  | >42 | 72 | 93 |  |
|  | <42 | 82 | 60 |  |
| **WHO grade** |  |  |  | <0.001 |
|  | II | 91 | 7 |  |
|  | III | 35 | 39 |  |
|  | IV | 28 | 107 |  |
| **IDH** |  |  |  | <0.001 |
|  | Mutant | 120 | 47 |  |
|  | Wildtype | 33 | 106 |  |
|  | NA | 1 | 0 |  |
| **1p/19q** |  |  |  | <0.001 |
|  | Codel | 62 | 1 |  |
|  | Non-codel | 90 | 149 |  |
|  | NA | 2 | 3 |  |

**Supplemental Table 5. The prognostic value of LARs in lower-grade gliomas in the CGGA dataset**

| **LARs** | **LGG** | | | | **LGG, IDH-wildtype** | | | | **LGG, IDH-mutant, 1p/19q non-codeletion** | | | | **LGG, IDH-mutant, 1p/19q codeletion** | | | |
| --- | --- | --- | --- | --- | --- | --- | --- | --- | --- | --- | --- | --- | --- | --- | --- | --- |
|  | **HR** | **95%CI** | | **p value** | **HR** | **95%CI** | | **p value** | **HR** | **95%CI** | | **p value** | **HR** | **95%CI** | | **p value** |
|  |  | **Low** | **High** |  |  | **Low** | **High** |  |  | **Low** | **High** |  |  | **Low** | **High** |  |
| **HDAC1** | 4.65 | 3.15 | 6.87 | 1.24E-14 | 3.28 | 1.58 | 6.8 | 1.42E-03 | 2.25 | 1.18 | 4.28 | 1.37E-02 | 5.97 | 0.69 | 51.6 | 1.05E-01 |
| **SIRT5** | 0.14 | 0.08 | 0.26 | 4.60E-10 | 0.3 | 0.13 | 0.69 | 4.40E-03 | 0.33 | 0.11 | 0.95 | 3.96E-02 | 1.33 | 0.03 | 56.11 | 8.81E-01 |
| **HDAC3** | 4.26 | 2.62 | 6.92 | 5.29E-09 | 2.48 | 1.19 | 5.19 | 1.55E-02 | 2.03 | 0.89 | 4.62 | 9.10E-02 | 2.52 | 0.23 | 27.23 | 4.47E-01 |
| **SIRT7** | 4.87 | 2.77 | 8.54 | 3.40E-08 | 3.52 | 1.67 | 7.43 | 9.59E-04 | 2.25 | 0.98 | 5.16 | 5.67E-02 | 1.29 | 0.14 | 11.71 | 8.23E-01 |
| **HAT1** | 3.87 | 2.32 | 6.47 | 2.37E-07 | 2.18 | 1.22 | 3.91 | 8.93E-03 | 2.94 | 1.35 | 6.36 | 6.36E-03 | 1.18 | 0.12 | 11.14 | 8.88E-01 |
| **HDAC7** | 2.66 | 1.83 | 3.85 | 2.81E-07 | 2.22 | 1.31 | 3.78 | 3.23E-03 | 1.4 | 0.78 | 2.51 | 2.55E-01 | 3.55 | 0.4 | 31.76 | 2.57E-01 |
| **HDAC4** | 0.46 | 0.34 | 0.62 | 4.08E-07 | 0.95 | 0.42 | 2.13 | 8.93E-01 | 0.68 | 0.44 | 1.06 | 8.88E-02 | 1.06 | 0.35 | 3.25 | 9.16E-01 |
| **SIRT3** | 0.26 | 0.15 | 0.44 | 1.06E-06 | 0.35 | 0.14 | 0.86 | 2.22E-02 | 0.57 | 0.27 | 1.2 | 1.39E-01 | 7.49 | 0.12 | 457.4 | 3.37E-01 |
| **ESCO2** | 1.73 | 1.38 | 2.16 | 1.36E-06 | 1.46 | 1.05 | 2.03 | 2.40E-02 | 1.52 | 1.07 | 2.16 | 1.98E-02 | 1.63 | 0.66 | 3.98 | 2.87E-01 |
| **HDAC11** | 0.58 | 0.44 | 0.78 | 2.55E-04 | 0.65 | 0.41 | 1.01 | 5.78E-02 | 0.81 | 0.49 | 1.31 | 3.88E-01 | 0.74 | 0.31 | 1.77 | 4.95E-01 |
| **LEF1** | 1.74 | 1.28 | 2.38 | 4.55E-04 | 1.8 | 1.23 | 2.64 | 2.68E-03 | 1.19 | 0.71 | 1.98 | 5.05E-01 | 2.33 | 0.53 | 10.22 | 2.63E-01 |
| **HDAC2** | 1.95 | 1.32 | 2.9 | 8.91E-04 | 1.41 | 0.9 | 2.21 | 1.31E-01 | 2.72 | 1.45 | 5.08 | 1.74E-03 | 2.05 | 0.43 | 9.87 | 3.68E-01 |
| **SIRT6** | 2.57 | 1.46 | 4.53 | 1.09E-03 | 2.64 | 1.24 | 5.59 | 1.16E-02 | 2.72 | 1.22 | 6.07 | 1.43E-02 | 1.49 | 0.2 | 11.04 | 6.95E-01 |
| **HDAC8** | 3.15 | 1.56 | 6.33 | 1.32E-03 | 2.73 | 1.08 | 6.92 | 3.42E-02 | 2.11 | 0.79 | 5.67 | 1.38E-01 | 1.32 | 0.11 | 16.39 | 8.30E-01 |
| **KAT2A** | 0.61 | 0.44 | 0.83 | 1.74E-03 | 1.46 | 0.86 | 2.49 | 1.62E-01 | 0.74 | 0.48 | 1.14 | 1.75E-01 | 1.29 | 0.33 | 4.98 | 7.12E-01 |
| **ESCO1** | 2.71 | 1.42 | 5.17 | 2.55E-03 | 2.05 | 0.81 | 5.17 | 1.29E-01 | 3.05 | 1.2 | 7.8 | 1.97E-02 | 1.49 | 0.15 | 14.46 | 7.30E-01 |
| **KAT6B** | 0.58 | 0.4 | 0.86 | 6.37E-03 | 0.79 | 0.4 | 1.56 | 4.92E-01 | 0.79 | 0.42 | 1.51 | 4.79E-01 | 0.8 | 0.14 | 4.72 | 8.05E-01 |
| **KAT2B** | 0.65 | 0.45 | 0.94 | 2.17E-02 | 1.14 | 0.67 | 1.93 | 6.25E-01 | 0.73 | 0.37 | 1.45 | 3.69E-01 | 2.69 | 0.38 | 19.09 | 3.23E-01 |
| **SIRT2** | 1.48 | 1.04 | 2.11 | 2.85E-02 | 1.42 | 0.82 | 2.46 | 2.15E-01 | 1.45 | 0.89 | 2.37 | 1.39E-01 | 2.94 | 0.8 | 10.84 | 1.06E-01 |
| **SIRT1** | 0.61 | 0.37 | 1.02 | 5.84E-02 | 1.23 | 0.43 | 3.48 | 7.00E-01 | 1.17 | 0.49 | 2.84 | 7.22E-01 | 1.14 | 0.21 | 6.2 | 8.76E-01 |
| **EP300** | 0.67 | 0.43 | 1.02 | 6.44E-02 | 1.43 | 0.73 | 2.8 | 2.97E-01 | 0.52 | 0.24 | 1.13 | 9.80E-02 | 1.68 | 0.26 | 10.86 | 5.85E-01 |
| **KAT7** | 0.78 | 0.55 | 1.12 | 1.85E-01 | 1.36 | 0.77 | 2.39 | 2.91E-01 | 1.37 | 0.71 | 2.64 | 3.43E-01 | 1.16 | 0.36 | 3.78 | 8.02E-01 |
| **KAT8** | 1.63 | 0.74 | 3.59 | 2.28E-01 | 1.93 | 0.66 | 5.69 | 2.32E-01 | 2.02 | 0.65 | 6.25 | 2.22E-01 | 2.87 | 0.12 | 69.21 | 5.16E-01 |
| **HDAC5** | 1.31 | 0.71 | 2.39 | 3.87E-01 | 0.78 | 0.35 | 1.71 | 5.33E-01 | 1.64 | 0.63 | 4.27 | 3.13E-01 | 4.62 | 0.58 | 37.03 | 1.50E-01 |
| **KAT5** | 0.73 | 0.33 | 1.62 | 4.34E-01 | 1.24 | 0.36 | 4.28 | 7.28E-01 | 2.63 | 0.83 | 8.36 | 1.01E-01 | 0.99 | 0.06 | 17.4 | 9.95E-01 |
| **SIRT4** | 1.17 | 0.77 | 1.79 | 4.58E-01 | 0.55 | 0.25 | 1.23 | 1.46E-01 | 0.96 | 0.57 | 1.63 | 8.81E-01 | 8.33 | 1.28 | 54.14 | 2.64E-02 |
| **HDAC9** | 1.1 | 0.82 | 1.49 | 5.11E-01 | 1.31 | 0.78 | 2.19 | 3.01E-01 | 0.95 | 0.64 | 1.43 | 8.20E-01 | 1.74 | 0.65 | 4.64 | 2.67E-01 |
| **HDAC6** | 0.88 | 0.56 | 1.38 | 5.72E-01 | 1.68 | 0.87 | 3.23 | 1.22E-01 | 1.15 | 0.59 | 2.22 | 6.81E-01 | 1.69 | 0.23 | 12.46 | 6.06E-01 |
| **CREBBP** | 0.91 | 0.61 | 1.36 | 6.51E-01 | 1.34 | 0.7 | 2.56 | 3.73E-01 | 1.01 | 0.53 | 1.92 | 9.84E-01 | 0.8 | 0.19 | 3.37 | 7.64E-01 |
| **SLC16A10** | 0.94 | 0.67 | 1.32 | 7.24E-01 | 0.81 | 0.54 | 1.23 | 3.32E-01 | 0.71 | 0.42 | 1.2 | 1.97E-01 | 2.24 | 0.75 | 6.69 | 1.47E-01 |
| **HDAC10** | 1.05 | 0.68 | 1.62 | 8.20E-01 | 1.7 | 0.88 | 3.31 | 1.17E-01 | 0.75 | 0.4 | 1.4 | 3.61E-01 | 1.92 | 0.54 | 6.85 | 3.14E-01 |
| **HNF1A** | 0.95 | 0.57 | 1.58 | 8.36E-01 | 0.68 | 0.33 | 1.39 | 2.89E-01 | 0.4 | 0.17 | 0.94 | 3.47E-02 | 3.53 | 0.38 | 32.44 | 2.65E-01 |
| **KAT6A** | 1 | 0.64 | 1.58 | 9.93E-01 | 1.6 | 0.87 | 2.92 | 1.31E-01 | 0.88 | 0.42 | 1.86 | 7.37E-01 | 1.08 | 0.16 | 7.51 | 9.38E-01 |

**Supplemental Table 6. The prognostic value of LARs in GBMs in the CGGA dataset**

|  | **GBM** | | | | **GBM, IDH-wildtype** | | | | **GBM, IDH-mutant** | | | |
| --- | --- | --- | --- | --- | --- | --- | --- | --- | --- | --- | --- | --- |
| **LARs** | **HR** | **95%CI** | | **p value** | **HR** | **95%CI** | | **p value** | **HR** | **95%CI** | | **p value** |
|  |  | **Low** | **High** |  |  | **Low** | **High** |  |  | **Low** | **High** |  |
| **KAT2B** | 0.7 | 0.53 | 0.92 | 1.15E-02 | 0.68 | 0.49 | 0.94 | 1.90E-02 | 0.74 | 0.4 | 1.4 | 3.59E-01 |
| **HDAC7** | 1.45 | 1.07 | 1.98 | 1.82E-02 | 1.63 | 1.05 | 2.52 | 3.01E-02 | 1.75 | 0.95 | 3.24 | 7.42E-02 |
| **HDAC8** | 1.49 | 0.94 | 2.38 | 9.14E-02 | 1.57 | 0.92 | 2.69 | 9.86E-02 | 1.31 | 0.51 | 3.35 | 5.76E-01 |
| **HDAC1** | 1.33 | 0.95 | 1.87 | 9.41E-02 | 1.16 | 0.72 | 1.89 | 5.41E-01 | 1.87 | 1.02 | 3.42 | 4.35E-02 |
| **HDAC3** | 1.43 | 0.92 | 2.25 | 1.15E-01 | 1.75 | 0.95 | 3.21 | 7.21E-02 | 1.16 | 0.56 | 2.41 | 6.94E-01 |
| **KAT5** | 0.63 | 0.35 | 1.14 | 1.25E-01 | 0.49 | 0.23 | 1.06 | 6.91E-02 | 0.73 | 0.23 | 2.29 | 5.89E-01 |
| **SIRT7** | 1.4 | 0.91 | 2.15 | 1.30E-01 | 1.32 | 0.79 | 2.21 | 2.84E-01 | 1.68 | 0.72 | 3.91 | 2.32E-01 |
| **LEF1** | 1.15 | 0.95 | 1.39 | 1.47E-01 | 1.34 | 1.06 | 1.69 | 1.62E-02 | 0.78 | 0.46 | 1.32 | 3.53E-01 |
| **HDAC4** | 0.81 | 0.6 | 1.1 | 1.80E-01 | 0.97 | 0.59 | 1.59 | 8.96E-01 | 0.51 | 0.29 | 0.91 | 2.38E-02 |
| **KAT2A** | 0.85 | 0.67 | 1.08 | 1.82E-01 | 0.91 | 0.68 | 1.21 | 5.01E-01 | 0.63 | 0.38 | 1.03 | 6.40E-02 |
| **HDAC11** | 0.85 | 0.67 | 1.08 | 1.84E-01 | 0.75 | 0.56 | 1.01 | 5.53E-02 | 1.25 | 0.76 | 2.04 | 3.79E-01 |
| **HAT1** | 1.22 | 0.9 | 1.66 | 1.95E-01 | 1.02 | 0.7 | 1.48 | 9.38E-01 | 2.07 | 1.12 | 3.82 | 2.00E-02 |
| **HDAC2** | 1.19 | 0.91 | 1.55 | 1.99E-01 | 1.06 | 0.75 | 1.51 | 7.41E-01 | 1.65 | 0.98 | 2.76 | 5.84E-02 |
| **SIRT2** | 0.86 | 0.68 | 1.09 | 2.10E-01 | 0.8 | 0.58 | 1.09 | 1.59E-01 | 0.86 | 0.53 | 1.4 | 5.43E-01 |
| **HNF1A** | 1.32 | 0.8 | 2.18 | 2.69E-01 | 1.16 | 0.65 | 2.07 | 6.22E-01 | 1.95 | 0.73 | 5.22 | 1.84E-01 |
| **SLC16A10** | 1.09 | 0.93 | 1.27 | 2.89E-01 | 1.07 | 0.9 | 1.28 | 4.15E-01 | 1.61 | 0.88 | 2.95 | 1.26E-01 |
| **CREBBP** | 0.85 | 0.63 | 1.15 | 2.94E-01 | 0.86 | 0.58 | 1.28 | 4.68E-01 | 0.73 | 0.41 | 1.29 | 2.79E-01 |
| **ESCO2** | 1.12 | 0.91 | 1.39 | 2.95E-01 | 1 | 0.76 | 1.33 | 9.81E-01 | 1.36 | 0.96 | 1.91 | 8.37E-02 |
| **KAT7** | 0.86 | 0.65 | 1.14 | 2.96E-01 | 0.76 | 0.53 | 1.08 | 1.28E-01 | 1.17 | 0.59 | 2.32 | 6.48E-01 |
| **KAT6B** | 0.89 | 0.68 | 1.17 | 4.14E-01 | 0.85 | 0.56 | 1.3 | 4.54E-01 | 0.67 | 0.33 | 1.37 | 2.73E-01 |
| **KAT6A** | 0.86 | 0.6 | 1.24 | 4.24E-01 | 0.8 | 0.51 | 1.25 | 3.23E-01 | 1 | 0.44 | 2.25 | 9.97E-01 |
| **KAT8** | 0.84 | 0.55 | 1.29 | 4.34E-01 | 1.05 | 0.6 | 1.83 | 8.73E-01 | 0.54 | 0.26 | 1.14 | 1.05E-01 |
| **HDAC10** | 1.12 | 0.83 | 1.5 | 4.68E-01 | 1.21 | 0.85 | 1.71 | 2.96E-01 | 0.86 | 0.47 | 1.57 | 6.14E-01 |
| **SIRT4** | 1.16 | 0.77 | 1.76 | 4.76E-01 | 0.98 | 0.6 | 1.61 | 9.37E-01 | 1.74 | 0.77 | 3.93 | 1.84E-01 |
| **SIRT5** | 0.86 | 0.54 | 1.39 | 5.45E-01 | 0.65 | 0.35 | 1.23 | 1.88E-01 | 1.3 | 0.58 | 2.92 | 5.21E-01 |
| **ESCO1** | 1.13 | 0.69 | 1.85 | 6.19E-01 | 0.77 | 0.42 | 1.39 | 3.80E-01 | 3.36 | 1.23 | 9.18 | 1.84E-02 |
| **SIRT6** | 1.09 | 0.77 | 1.54 | 6.29E-01 | 1.06 | 0.71 | 1.59 | 7.71E-01 | 1.16 | 0.57 | 2.37 | 6.74E-01 |
| **HDAC5** | 0.92 | 0.63 | 1.34 | 6.48E-01 | 0.83 | 0.48 | 1.45 | 5.16E-01 | 0.79 | 0.27 | 2.3 | 6.64E-01 |
| **HDAC6** | 1.07 | 0.76 | 1.51 | 6.99E-01 | 1.1 | 0.71 | 1.71 | 6.63E-01 | 1.01 | 0.57 | 1.8 | 9.64E-01 |
| **SIRT1** | 0.95 | 0.68 | 1.32 | 7.54E-01 | 0.9 | 0.58 | 1.41 | 6.47E-01 | 1.02 | 0.52 | 1.98 | 9.59E-01 |
| **HDAC9** | 1.04 | 0.81 | 1.34 | 7.64E-01 | 0.96 | 0.72 | 1.29 | 7.87E-01 | 1.37 | 0.81 | 2.31 | 2.39E-01 |
| **SIRT3** | 0.98 | 0.67 | 1.43 | 9.21E-01 | 1.13 | 0.72 | 1.78 | 6.01E-01 | 0.72 | 0.37 | 1.42 | 3.47E-01 |
| **EP300** | 0.99 | 0.72 | 1.36 | 9.70E-01 | 1.02 | 0.69 | 1.51 | 9.18E-01 | 0.9 | 0.49 | 1.67 | 7.44E-01 |

| **Supplemental Table 7. Different CNV patterns of HDAC1 and SIRT2 between 1p/19q codeletion and non-codeletion** | | | | |
| --- | --- | --- | --- | --- |
|  |  | **1p/19q Non-codeletion** | **1p/19q Codeletion** | **p value** |
| **Total case** |  | 449 | 149 |  |
| **SIRT2(19q)** |  |  |  | p<0.001 |
|  | Loss | 59 | 149 |  |
|  | Diploid | 317 | 0 |  |
|  | Gain | 73 | 0 |  |
| **HDAC1(1p)** |  |  |  | p<0.001 |
|  | Loss | 27 | 149 |  |
|  | Diploid | 381 | 0 |  |
|  | Gain | 41 | 0 |  |
